# Changes in Neuroimmune and Neuronal Death Markers after Adolescent Alcohol Exposure in Rats are Reversed by Donepezil

**DOI:** 10.1038/s41598-019-47039-1

**Published:** 2019-08-20

**Authors:** H. S. Swartzwelder, Kati L. Healey, Wen Liu, Kira Dubester, Kelsey M. Miller, Fulton T. Crews

**Affiliations:** 1 Department of Psychiatry and Behavioral Sciences, Duke University Medical Center, Durham, N.C. USA; 2 Bowles Center for Alcohol Studies, University of North Carolina, Chapel Hill, N.C. USA

**Keywords:** Neuroscience, Neurology

## Abstract

Adolescent intermittent ethanol (AIE) exposure diminishes neurogenesis and dendritic spine density in the dentate gyrus. The cholinesterase inhibitor, donepezil (Aricept), reverses AIE effects on dendritic spines, possibly by interacting with inflammatory and/or epigenetic mediators after AIE exposure. This study tests the hypothesis that donepezil reverses AIE-induced neuroimmune, and epigenetic changes in the adult dentate gyrus. Adolescent Sprague-Dawley male rats (PD30-43) were given 10 intermittent, intragastric doses of ethanol (5.0 g/kg) or isovolumetric water (AIW). Twenty-one days later half of the animals from each group were treated with either donepezil or isovolumetric water (i.g.) once daily for four days. Two hours after the last donepezil or water dose animals were sacrificed and brains prepared for immunohistochemical analyses. AIE reduced immunoreactivity for doublecortin (DCX) and increased immunoreactivity for activated caspase-3 and death receptor-3 in adulthood, suggesting an enduring attenuation of neurogenesis and an increase in progenitor death. These effects were reversed by donepezil treatment in adulthood. AIE also increased immunoreactivity for the inflammatory signaling molecules HMGB1 and RAGE, as well as the activated phosphorylated transcription factor pNFκB p65, and the gene silencing marker dimethylated histone H3K9. All of these AIE effects were also reversed by donepezil, with the exception of HMGB1.

## Introduction

Adolescence is a time of significant behavioral and neurological change, and represents a distinct developmental epoch. This period begins with the onset of puberty, spans the teen years, and proceeds well into the 20 s, especially in men^1^. This stage of late brain development is critically important for an individual’s ability to function effectively in the adult world. Whatever the specific nomenclature used to describe this developmental period, adolescence/young adulthood has become the subject of intense investigation^2–4^. In both humans and rodents, adolescents and young adults respond differently to acute ethanol^3,5–8^ and are more sensitive to the negative effects of repeated ethanol exposure that persist into adulthood (see^2,4^). It is also known that early age drinking onset is associated with elevated alcohol abuse liability in adulthood^9,10^. Thus, the distinctive characteristics of the adolescent brain may contribute to the high rate of drinking among some adolescents and young adults, and render them vulnerable to permanent neurological changes as a result of drinking during those periods. In support of this, preclinical rodent research shows AIE-induced structural and morphometric changes to the hippocampus in adulthood^4,11^. Reversal of these permanent neurological changes could lead to a reduction in adulthood alcohol use disorders. One promising treatment is donepezil (Aricept), a cholinesterase inhibitor, which has recently been shown to reverse persistent AIE-induced hippocampal changes in adulthood^12^.

Aforementioned, the adolescent brain is uniquely sensitive to alcohol-induced insult, particularly in the hippocampal formation^13,14^. Several magnetic resonance imaging studies of human adolescent alcohol users have shown hippocampal volume decreases associated with their alcohol use^15–17^. Studies in human adolescents are not able to distinguish whether decreased hippocampal volume represents a consequence of alcohol use, exposure to other factors^18^, or are a pre-existing condition promoting adolescent alcohol use, although preclinical studies have reported reduced hippocampal volume after AIE^19^. Adolescent hippocampal neurogenesis provides a robust indicator of neuron formation and is greater in adolescence and declines in middle age. Interestingly, AIE treatment of adolescents causes a persistent loss of neurogenesis into adulthood whereas identical adult intermittent treatment (CIE) causes a transient loss of neurogenesis that reverses in abstinence^20,21^. A persistent loss of hippocampal neurogenesis could contribute to the loss in hippocampal volume in alcoholism, although other mechanisms are likely involved as well.

Hippocampal neural progenitor cells (NPC) have been linked to a number of functions including, memory, learning, depression and anxiety^22,23^. Adolescent rats have higher levels of hippocampal neurogenesis than adult rats, and neurogenesis decreases across the period from adolescence to adulthood^24^. Reduced hippocampal volume and lower levels of hippocampal neurogenesis may underlie the symptoms and etiology of affective disorders that typically manifest during adolescence^25–28^. We have found that AIE causes an increase in the sensitivity of adult animals to the effects of acute ethanol on spatial learning in the radial arm maze^29,30^. However, most studies have found no marked effects of AIE on simple spatial learning tasks in the absence of acute ethanol or other challenges^29,31,32^ – although we have shown modest but significant deficits on an object recognition task that simultaneously assesses both spatial and temporal object recognition^33^. These deficits may be attributed to the changes induced by AIE in hippocampal structure and function. Studies have shown evidence of AIE-induced neuronal loss in area CA1^11^ and reduced neurogenesis in the granule cell layer of the dentate gyrus^34^ in adulthood, well after the termination of AIE exposure. In addition, AIE has been shown to alter long-term potentiation (LTP)^11^, astrocyte activation^35^, *N*-methy-D-aspartate receptor-mediated current amplitudes^36^, and dendritic spine density^12^ in the hippocampal formation in adulthood. An AIE-induced reduction of dendritic spine density was observed in the dentate gyrus, and was reversed by sub-chronic systemic treatment with donepezil in adulthood^12^. Reductions of neurogenesis have also been observed after AIE in this region^34^, but the impact of donepezil on AIE reduced neurogenesis had not been studied previously.

Donepezil (Aricept) is a selective, reversible acetylcholinesterase inhibitor in current clinical use to ameliorate memory-related cognitive deficits. In rodents donepezil pre-treatment protects against the heightened vulnerability to hippocampally-mediated learning deficits after cholinergic lesions^37,38^, and against increases of caspase-3 (a marker of synaptic and neuronal degeneration)^39^ in the hippocampus. One prominent hypothesis for the effects of AIE on hippocampal structure and function is a possible induction of inflammatory mechanisms that could be neurotoxic^40^. Acetylcholine has been shown to inhibit inflammatory signaling and the production of proinflammatory cytokines^41^. Thus, it is possible that post-AIE treatment with donepezil could reverse hippocampal deficits through a cholinergically mediated anti-inflammatory mechanism. Alternatively, donepezil has been found to directly inhibit microglia through a non-cholinergic mechanism^42^. Histone deacetylase inhibitors alter epigenetic gene silencing, are anti-inflammatory and have been found to reverse AIE induced inhibition of neurogenesis and restore brain-derived neurotropic factor (BDNF) expression^14^. For these reasons the present experiments were designed to assess the effects of AIE on neurogenesis and neural survival in the dentate gyrus and to determine if changes in those parameters are accompanied by changes in the expression of inflammatory and epigenetic markers and/or reversed by sub-chronic systemic treatment with donepezil.

## Materials and Methods

### Animals and ethanol exposure

The procedures used in this study were conducted in accordance with the guidelines of the American Association for the Accreditation of Laboratory Animal Care and the National Research Council’s Guide for Care and Use of Laboratory Animals and were approved by the Duke University Institutional Animal Care and Use Committee.

Adolescent male Sprague-Dawley rats [Charles River, Raleigh, NC; postnatal day (PND) 25] were allowed 5 days to acclimate to the vivarium on a reversed 12-hour light: 12-hour dark cycle (ights on from 9 pm to 9 am). Animals were double housed with ad libitum access to food and water. Animals underwent either AIE or water (AIW) exposure beginning on PND30 for a series of 10 doses over the course of 16 days using a two day on, one day off, two days on, two days off schedule. 5.0 g/kg ethanol (35% v/v in water at 18.12 ml/kg; VWR, Suwanee, GA) or isovolumetric water was administered by intragastric gavage (i.g.). This ethanol dose was selected to produce blood ethanol concentrations consistent with those achieved by human adolescents during binge drinking episodes^43^. In order to minimize stress to experimental animals we routinely test sentinel animals in our laboratory for BECs using the gavage doses in our model. We have found that rats of the age, sex, and strain used in this study, receiving 5 g/kg ethanol (i.g.), achieved mean blood ethanol concentrations of 199.7 mg/dl (±19.9) 60 minutes after the first dose, and 172.8 (±13.3) 60 minutes after the last dose^11^. Those blood ethanol concentrations are consistent with those achieved across multiple years in our earlier studies^44^. Following twenty-one days washout after the last ethanol exposure, i.e. PND69, animals were treated with either 2.5 mg/kg donepezil (DZ), 1.88 ml/kg, i.g., in water) or isovolumetric water (vehicle; VEH) daily, for four days, and were sacrificed two hours after the last donepezil dose on PND72. The groups are as follows: AIW/VEH (n = 6), AIW/DZ (n = 6), AIE/VEH (n = 7), AIE/DZ (n = 7). One consideration with involuntary ethanol exposure modes is the stress that they may induce. We have chosen i.g. administration because i.p. injections and vapor exposure, also produce stress in rodents and because humans consume ethanol through the gastric route. We endeavor to make our exposure model as translationally relevant as possible. Moreover, our experimental design equalizes the stress across treatment groups such that all animals receive the same number of gavage exposures regardless of treatment group. Still, results must be interpreted in light of possible stress effects.

### Tissue collection and preparation

Rats were deeply anesthetized with isoflurane and perfused transcardially with 0.1 M phosphate-buffered saline (PBS, pH 7.4) followed by 4% paraformaldehyde (in 0.1 M phosphate buffer, pH 7.4). Next, the brains were removed and post-fixed for 24 hours in 4% paraformaldehyde at 4 °C. Coronal sections (40 μm) were sliced in 1:12 series after cryoprotection with 30% sucrose. Every twelfth section was used for each of the antigens in Table 1.

**Table 1:** Primary antibodies for the present study.

Antibodies	Isotype	Source/Purification	Dilution	Source
DCX	goat IG	Polyclonal	1:200	Santa Cruz Biotechnology, Inc., Dallas, TX (sc-8066)
Cleaved Caspase-3 (Asp175)	Rabbit IgG	Polyclonal	1:1200	Cell signaling technology, Danvers, MA, USA (#9661)
pNFkB p65 (phospho S536)	Rabbit IgG	Polyclonal	1:1000	Abcam Inc., Cambridge, MA, USA (ab86299)
TNFRSF25/DR3 antibody	Rabbit IgG	polyclonal	1:200	Lifespan biosciences, Inc., Seattle, WA, USA (LS-B7731)
Anti-Histone H3 (di methyl K9)	Mouse IgG2a	monoclonal	1:800	Abcam Inc., Cambridge, MA, USA (ab1220)
RAGE	Rabbit IgG	polyclonal	1:1000	Abcam Inc., Cambridge, MA, USA (ab3611)
HMGB1	Rabbit IgG	polyclonal	1:2000	Abcam Inc., Cambridge, MA, USA (ab18256)

### Histology procedure

The sections were incubated in 0.6% H_2_O_2_ for 30 minutes to remove endogenous peroxidase activity, and blocked in 5% goat serum or rabbit serum (0.2% Triton X-100) for 1 hour at room temperature. All primary antibodies were used at different dilutions (see Table 1) overnight at 4 °C. Sections were rinsed in phosphate-buffered saline, and incubated with biotinylated secondary goat anti-rabbit or rabbit anti-goat antibody (1:200, Vector Laboratories, Burlingame, CA, USA) for 1 hour at room temperature. Avidin-biotin-peroxidase complex (ABC Elite Kit, Vector Laboratories) was later added for one hour at room temperature. The positive expression was visualized using nickle-enhanced diaminobenzidine (DAB).

### Quantification

Bioquant Nova Advanced Image Analysis (R&M Biometric, Nashville, TN) was used to capture and analyze images^21^, which were captured using an Olympus BX50 Microscope and Sony DXC-390 video camera linked to a computer. In the hippocampus, positive (immunoreactive; IR) cells (activated caspase-3+, TNFRSF25/DR3+, phosphorylated nuclear factor kappa-light-chain enhancer of activated B (*p*NFκB)*p*65+, phosphoinositide 3-kinase (PI3K)+, HM3K9me2+, receptor for advanced glycation endproducts (RAGE)+, and HMGB1 + IR) were profile counted in the granule cell layer of the dorsal dentate gyrus (DG)(Bregma from −2.30 to −4.52 mm) and expressed as cells per square millimeter with both sides of 3–5 section per animals. The average value per mm^2^ was used for analyses. Immuno-positive cell are counted using a modified stereology counting methods^21^. In the case of DCX, neuronal quantification can be difficult. DCX is a cytoskeletal protein that is commonly used to provide an index of neurogenesis because it is only expressed in newly formed neurons^45^. However, DCX stains the entire progenitor cell resulting overlap that makes individual cells difficult to distinguish and count. Thus, for DCX + IR the granule cell layer of the hippocampal DG was outlined and immunopositive pixel density was measured for the outlined area (pixels/mm^2^). One subject was removed from the DCX analysis (AIE/VEH) because the tissue was damaged beyond quantification.

### Data analysis

For the effect of AIE exposure and donepezil treatment on positive cells and pixel values, analysis of variance (ANOVA) was used to test statistical significance, followed by Fisher’s LSD tests to assess the significance of planned comparisions based on our hypothesis that donepezil will reverse the effects of AIE on neuronal and inflammatory markers. In the event of hypothesized ordinal interactions, where there is no effect at one level of the dependent variable but an effect at the other level, there are often not statistically significant interactions. In such instances post-hoc comparisons are the statistically preferred way to determine effects, and we analyzed such outcomes accordingly. Significant differences were concluded when *p* < 0.05. Planned pairwise comparisions were performed in the presence of ordinal ineractions.

## Results

### AIE exposure reduces hippocampal dentate gyrus neurogenesis and the AIE deficit is reversed by Donepezil

**Figure 1:**
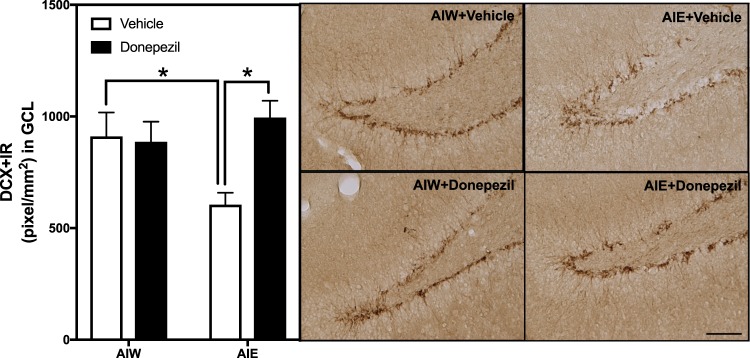
AIE-Induced Reduction of Doublecortin Immunoreactivity in the Dentate Gyrus is Reversed by Donepezil. Left Panel: Mean (+SEM) pixels/mm^2^ of DCX immunoreactivity in the granule cell layer of the dentate gyrus from dorsal hippocampus in rat brains exposed to AIE or AIW, and treated in adulthood with donepezil (filled bars) or the control vehicle (open bars). AIW/VEH n = 6, AIW/DZ n = 6, AIE/VEH n = 7, AIE/DZ n = 6 *p < 0.05 in *post hoc* analyses. Right Panels: Representative photomicrographs of DCX + IR cells in the dentate gyrus of adult animals exposed to control vehicle in adolescence and in adulthood (AIW + Vehicle), AIE during adolescence and control vehicle in adulthood (AIE + Vehicle), control vehicle in adolescence and donepezil in adulthood (AIW + Donepezil), or AIE during adolescence and donepezil in adulthood (AIE + Donepezil) (Immunohistochemical staining, Bar scale = 25 μm).

The hippocampal dentate gyrus continues neurogenesis, i.e. the formation of new neurons, into adulthood, although neurogenesis in adolescence is high and declines with increasing age in adulthood^46–48^. We used doublecortin, a protein only expressed in developing neuroprogenitor cells, but not mature neurons, as a marker for neurogenesis in AIE exposed animals compared to controls in adulthood, as has been reported previously^34,49^. We found a significant effect of the interaction between AIE and donepezil for DCX + IR expression [*F*_(1,21)_ = 6.39, *p* < 0.02, Fig. 1]. *Post-hoc* analysis revealed AIE exposure reduced DCX + IR expression (34% ±6, *p* < 0.02) in the dentate gyrus, thus replicating the previous findings. Following AIE and a 21-day washout period with no treatment, a four-day treatment with donepezil increased DCX + IR expression (65% ±12, *p* < 0.01, Fig. 1] compared to AIE exposed animals treated with the donepezil vehicle. Thus, AIE exposure causes a persistent loss of DCX + IR neurogenesis that can be reversed in adulthood by donepezil treatment.

### AIE exposure increases cell death machinery in the hippocampal dentate gyrus and is reversed by Donepezil

**Figure 2:**
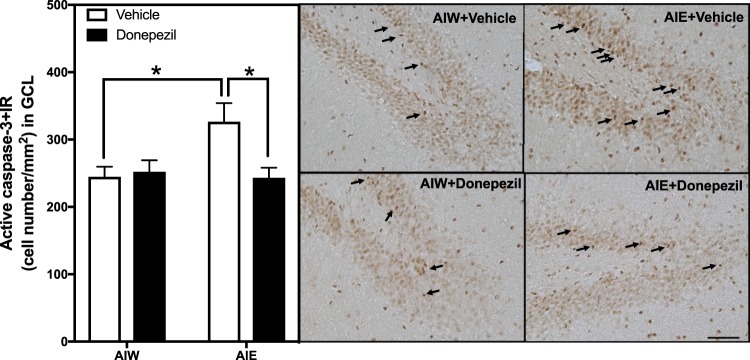
AIE-Induced Increase of Active Caspase-3 Immunoreactivity in the Dentate Gyrus is Reversed by Donepezil. Left Panel: Mean (+SEM) number of caspase-3 + IR positive cells/mm^2^ in the granule cell layer of the dentate gyrus from dorsal hippocampus in rat brains exposed to AIE or AIW, and treated in adulthood with donepezil (filled bars) or the control vehicle (open bars). AIW/VEH n = 6, AIW/DZ n = 6, AIE/VEH n = 7, AIE/DZ n = 7 *p < 0.05 in *post hoc* analyses. Right Panels: Representative photomicrographs of caspase-3 + IR cells in the dentate gyrus of adult animals exposed to control vehicle in adolescence and in adulthood (AIW + Vehicle), AIE during adolescence and control vehicle in adulthood (AIE + Vehicle), control vehicle in adolescence and donepezil in adulthood (AIW + Donepezil), or AIE during adolescence and donepezil in adulthood (AIE + Donepezil). Arrows identify active caspase-3 + IR cells (Immunohistochemical staining, Bar scale = 25 μm).

**Figure 3:**
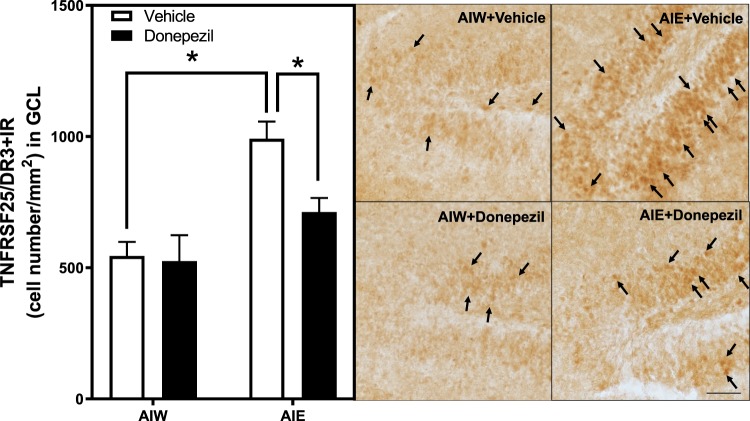
AIE-Induced Increase of Death Receptor-3 Immunoreactivity in the Dentate Gyrus is Reversed by Donepezil. Left Panel: Mean (+SEM) number of DR3 + IR positive cells/mm^2^ in the granule cell layer of the dentate gyrus from dorsal hippocampus in rat brains exposed to AIE or AIW, and treated in adulthood with donepezil (filled bars) or the control vehicle (open bars). AIW/VEH n = 6, AIW/DZ n = 6, AIE/VEH n = 7, AIE/DZ n = 7 *p < 0.05 in *post hoc* analyses. Right Panels: Representative photomicrographs of DR3 + IR cells in the dentate gyrus of adult animals exposed to control vehicle in adolescence and in adulthood (AIW + Vehicle), AIE during adolescence and control vehicle in adulthood (AIE + Vehicle), control vehicle in adolescence and donepezil in adulthood (AIW + Donepezil), or AIE during adolescence and donepezil in adulthood (AIE + Donepezil). Arrows identify DR3 + IR cells (Immunohistochemical staining, Bar scale = 25 μm).

The observed AIE-induced deficits on hippocampal neurogenesis indicate increased cell death and likely an induction of apoptosis mechanisms. To investigate this, we probed for differences in cell death machinery, activated caspase-3 and death receptor-3 (DR3). Caspase-3 is an executioner caspase, activated by cleavage, resulting in caspase protease activity which leads to apoptosis and other forms of cell death. We used an antibody that labeled cleaved activated caspase 3 to identify dying cells. Previous studies have linked neuroimmune activation by AIE to reduced neurogenesis and increased cleaved-caspase-3 + IR in the dentate gyrus^50^. In this study we found a significant AIE x donepezil interaction for activated caspase-3 + IR [*F*_(1,22)_ = 5.21, *p* < 0.05, Fig. 2]. Post-hoc analyses indicated AIE exposure increased activated caspase-3 (33% ±11, *p* < 0.01) and treatment with donepezil reversed this increase (*p* < 0.01). DR3 is a member of the TNF receptor superfamily, e.g. TNFSR25, that are known to activate caspase mediated cell death cascades^51^. AIE exposure also significantly increased DR3 + IR expression compared to control animals (82% ±12), there was a main significant effect of AIE [*F*_(1,22)_ = 20.9, *p* < 0.01, Fig. 3], consistent with neuroimmune death receptors initiating cell death caspase cascades in neuroprogenitors. Interestingly, treatment with donepezil reversed the effects of AIE on DR3 + IR expression (*post-hoc* Fisher LSD, *p* < 0.01). Although there was no significant interaction between AIE and donepezil [*F*_(1,22)_ = 1.58, p = 0.22, Fig. 3] in this instance, we utilized this post-hoc comparison because of the presence of the hypothesized ordinal interaction. These findings suggest AIE exposure increases DR3-caspase mediated neuroprogenitor cell death that is reversed by donepezil in adulthood.

### AIE exposure alters neuroimmune function and epigenetic plasticity in the hippocampal dentate gyrus and Donepezil reverses these alterations

**Figure 4:**
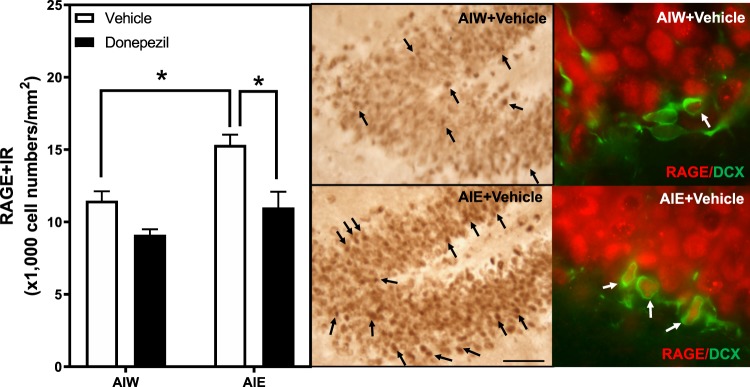
AIE-Induced Increase of RAGE Receptor Immunoreactivity in the Dentate Gyrus is Reversed by Donepezil. Left Panel: Mean (+SEM) number of RAGE + IR positive cells/mm^2^ in the granule cell layer of the dentate gyrus from dorsal hippocampus in animals exposed to AIE or AIW, and treated in adulthood with donepezil (filled bars) or the control vehicle (open bars). AIW/VEH n = 6, AIW/DZ n = 6, AIE/VEH n = 7, AIE/DZ n = 7 *p < 0.05 in *post hoc* analyses. Middle Panels: Representative photomicrographs of RAGE + IR cells in the dentate gyrus of adult animals exposed to control vehicle in adolescence (AIW + Vehicle) or AIE during adolescence (AIE + Vehicle). Arrows identify RAGE + IR cells (Immunohistochemical staining, Bar scale = 25 μm). Right Panels: Elevated magnification images of the dentate granule cell region from animals exposed to control vehicle (AIW + Vehicle) or AIE (AIE + Vehicle) during adolescence, co-stained for RAGE and DCX. Arrows identify RAGE+/DCX + IR cells (Original magnification 100×).

**Figure 5:**
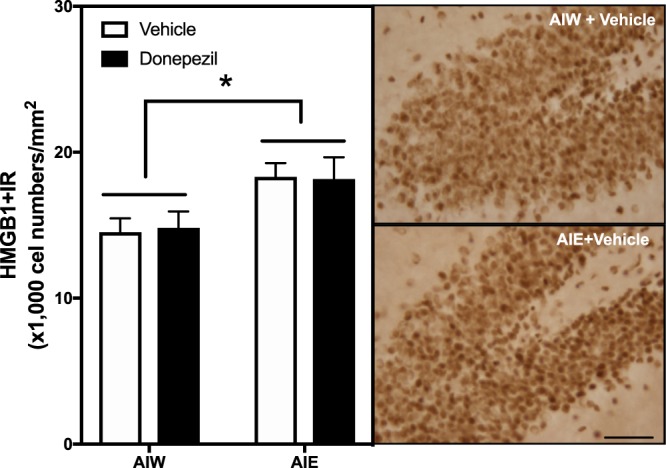
AIE-Induced Increase of HMGB1 Immunoreactivity in the Dentate Gyrus is not Reversed by Donepezil. Left Panel: Mean (+SEM) number of HMGB1 + IR positive cells/mm^2^ in the granule cell layer of the dentate gyrus from dorsal hippocampus in rat brains exposed to AIE or AIW, and treated in adulthood with donepezil (filled bars) or the control vehicle (open bars). AIW/VEH n = 6, AIW/DZ n = 6, AIE/VEH n = 7, AIE/DZ n = 7 *p < 0.05. Right Panels: Representative photomicrographs of HMGB1 + IR cells in the dentate gyrus of adult animals exposed to control vehicle in adolescence (AIW + Vehicle) or AIE in adolescence (AIE + Vehicle) (Immunohistochemical staining, Bar scale = 25 μm).

Previous studies have also found that AIE exposure causes a persistent increase in hippocampal expression of proinflammatory Toll-like receptors (TLR), the proinflammatory cytokine TNF-α and high mobility group box 1 (HMGB1), as well as the transcriptionally active subunit phosphorylated (activated) NFκB (pNFκB p65) common to proinflammatory signaling^52,53^. The persistence of neuroimmune signaling after AIE may be related to the persistent increases in adult hippocampal HMGB1, which can activate TLR and the receptor for advanced glycation end-products (RAGE). Studies have found that systemic treatment with endotoxin, i.e. lipopolysaccharide (LPS), increases hippocampal proinflammatory genes and reduces neurogenesis, mimicking AIE-induced loss of neurogenesis. Further, RAGE activation, among other mechanisms, increases NFκb activation and leads to increases in transcription of proinflammatory cytokines^54,55^. The anti-inflammatory drug indomethacin and voluntary exercise each prevent AIE-induced loss of neurogenesis and increased pNFκBp65 + IR^50,56^. To investigate potential mechanisms of neuroimmune activation we measured RAGE + IR and HMGB1, an agonist that activates RAGE. AIE exposure significantly increased RAGE + IR expression (34%±6, *p* < 0.01), and treatment with donepezil reversed this increase (*p* < 0.05, Fig. 4). ANOVA revealed that there was a significant main effect of AIE *F*_(1,22)_ = 13.40, *p* < 0.01], but no AIE × donepezil interaction [*F*_(1,22)_ = 1.58, p = 0.22]. AIE exposure also increased HMGB1 + IR expression (24% ± 6, Fig. 5), and there was a main effect of AIE [*F*_(1,22)_ = 13.40, *p* < 0.01], however donepezil had no effect on HMGB1 + IR [*F*_(1,22)_ = 0.004, *p* = 0.95] and there was no interaction [*F*_(1,22)_ = 0.04, p = 0.85]. Similar to other neuroimmune receptors, RAGE is both a target and an activator of NFκB cascades leading to proinflammatory gene induction. Thus, donepezil might act in part by blocking neuroimmune gene induction.

**Figure 6:**
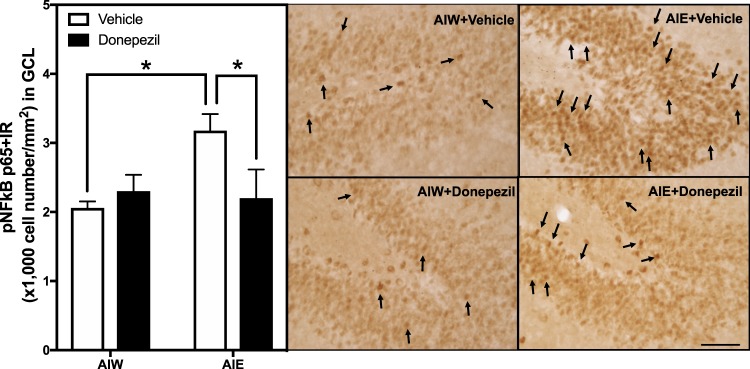
AIE-Induced Increase of *p*NFκB *p*65 Immunoreactivity in the Dentate Gyrus is Reversed by Donepezil. Left Panel: Mean (+SEM) number of pNFkB IR positive cells/mm^2^ in the granule cell layer of the dentate gyrus from dorsal hippocampus in rat brains exposed to AIE or AIW, and treated in adulthood with donepezil (filled bars) or the control vehicle (open bars). AIW/VEH n = 6, AIW/DZ n = 6, AIE/VEH n = 7, AIE/DZ n = 7 *p < 0.05 in *post hoc* analyses. Right Panels: Representative photomicrographs of *p*NFκB + IR cells in the dentate gyrus of adult animals exposed to control vehicle in adolescence and in adulthood (AIW + Vehicle), AIE during adolescence and control vehicle in adulthood (AIE + Vehicle), control vehicle in adolescence and donepezil in adulthood (AIW + Donepezil), or AIE during adolescence and donepezil in adulthood (AIE + Donepezil). Arrows identify pNFkBp65 + IR cells (Immunohistochemical staining, Bar scale = 25 μm).

As mentioned above, AIE increases NFκB transcription of proinflammatory cytokine messinger RNA (mRNA). In this study we determined levels of adult hippocampal dentate gyrus pNFκB p65 + IR, the transcriptionally active form of NFκB, as an indicator of increased transcription. Our previous studies found that AIE combined with indomethacin or exercise blocked AIE-induced inhibition of neurogenesis^57^. In the current study we investigated the ability of donepezil to reverse AIE-induced inhibition of neurogenesis. We also replicated the previously reported persistent loss of DCX, and the increased adult pNFκB p65 + IR after AIE (an index of neuroimmune activation) to determine the effect of adult donepezil treatment on these AIE-induced effects^52,53^. Consistent with previous research, we found a significant effect of the interaction between AIE and donepezil for increased *p*NFκB *p*65 + IR [54% ±12, interaction *F*_(1,22)_ = 4.56, *p* < 0.05, Fig. 6]. *Post-hoc* analysis showed an AIE-induced increase in *p*NFκB *p*65 + IR expression (*p* < 0.02), and significantly lower expression in animals that had received AIE followed by donepezil, compared to those that received AIE alone (*p* < 0.02), indicating that donepezil reversed the impact of AIE on *p*NFκB *p*65 + IR expression. Interestingly, the prevention of AIE-induced loss of neurogenesis by exercise also prevented of AIE-induced proinflammatory gene induction, but did not prevent the AIE-increase in adult HMGB1^50,57^. These findings indicate that donepezil reversed AIE-induced proinflammatory *p*NFκB signaling as well as the loss of neurogenesis and increased cell death.

**Figure 7:**
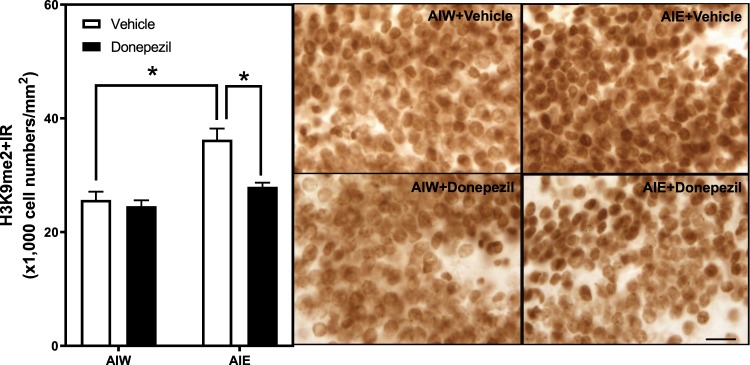
AIE-Induced Increase of Dimethlyated H3K9 Immunoreactivity in the Dentate Gyrus is Reversed by Donepezil. Left Panel: Mean (+SEM) number of H3K9me2 + IR positive cells/mm^2^ in the granule cell layer of the dentate gyrus from dorsal hippocampus in animals exposed to AIE or AIW, and treated in adulthood with donepezil (filled bars) or the control vehicle (open bars). AIW/VEH n = 6, AIW/DZ n = 6, AIE/VEH n = 7, AIE/DZ n = 7 *p < 0.05 in *post hoc* analyses. Right Panels: Representative photomicrographs of H3K9me^2^ + IR cells in the dentate gyrus of adult animals exposed to control vehicle in adolescence and in adulthood (AIW + Vehicle), AIE during adolescence and control vehicle in adulthood (AIE + Vehicle), control vehicle in adolescence and donepezil in adulthood (AIW + Donepezil), or AIE during adolescence and donepezil in adulthood (AIE + Donepezil) (Immunohistochemical staining, Bar scale = 10 μm).

Although many studies have linked AIE inhibition of neurogenesis to proinflammatory gene induction and increased cell death, it has also been linked to decreases in brain-derived neurtropic factor (BDNF), a hippocampal trophic factor^58^. BDNF protein and exon IV mRNA levels were reduced in the CA1 and CA3 after AIE, and treatment with the histone deacetylase inhibitor, trichostatin A (TSA), reversed AIE-induced inhibition of both neurogenesis and BDNF, but no measures of neuroimmune gene changes have previously been made. To investigate potential epigenetic mechanisms of the donepezil-induced reversal of AIE effects, we assessed histone H3 lysine 9 dimethylation (H3K9me2). AIE-induced increases in H3K9me2 in the central amygdala have been linked to epigenetic remodeling that may be associated with adult anxiety^58^. We report here, consistent with the amygdala findings, that AIE exposure increased H3K9me2 + IR expression (41% ± 8, *p* < 0.01, Fig. 7). There was a significant main effect of AIE [*F*_(1,22)_ = 24.88, *p* < 0.01], and AIE×donepezil interaction [*F*_(1,22)_ = 6.55, *p* < 0.02]. *Post-hoc* analyses revealed that treatment with donepezil reversed these AIE-induced effects on H3K9me2 + IR (*p* < 0.01). These findings are consistent with the hypothesis that donepezil reversal of AIE loss of neurogenesis is linked to a reversal of both neuroimmune and epigenetic changes that persist into adulthood. The reversal of the AIE-induced loss of neurogenesis suggests persistent down regulation of neurogenesis that can be reversed, rather than a permanent loss of neural progenitor cells and stem cells that cannot be reversed.

## Discussion

The principle findings of this study are that AIE caused a persistent reduction in adult doublecortin staining, and promoted cell death in the dentate gyrus of the hippocampal formation. Both of these effects were reversed by sub-chronic treatment with donepezil in adulthood. Specifically, immunoreactivity for doublecortin (IR + DCX; a proxy for neurogenesis) was decreased, and immunoreactivity for activated caspase-3 (reflecting apoptotic cell death) and death receptor 3 (DR3-TNFRSF25; caspase cell death cascade signaling) were increased in adulthood after AIE. All of those changes were reversed to control levels by treatment with donepezil. Similarly, AIE elevated both neuroimmune and epigenetic markers in the dentate gyrus, and those effects were also reversed by donepezil treatment in adulthood, with the exception of the increase in high mobility group box 1 (HMGB1). These findings suggest AIE increases neuroimmune gene expression that persistently blunts neurogenesis through increased progenitor cell death. Because of the large number of dependent variables measured in this study, and the previous studies on which these experiments were based, only male rats were used. Studies are underway to compare sex as a biological variable on the outcome measures we identified as vulnerable to AIE and donepezil reversal. Such comparative studies will enable a broader interpretation of the data than is possible with present findings. In addition, it is notable that we chose to use intragastric gavage as the protocol for ethanol administration in order to be able to achieve blood ethanol concentrations consistent with binge-like drinking episodes in humans. We recognize that the stress associated with intragastric gavage represents a limitation of the present study, and that there are self-administration protocols that presumably produce less stress (though producing lower blood ethanol concentrations). However, we endeavored to control for the stress by including an appropriate water gavage group, and we believe that the consistency of exposure afforded by the gavage method conveys dose consistency that enhances the interpretability of the findings.

Among the findings in this report several have mechanistic implications. Specifically, AIE increased immunoreactivity for the receptor of advanced glycation endproduct (RAGE; pro-inflammatory gene activation), the pNFκB p65 transcriptionally active protein (cytokine production, immune response regulation^59^), and dimethylated histone H3K9 (H3K9me2, an epigenetic mark for repressed transcription). Sub-chronic treatment with donepezil reversed these AIE-induced increases to control levels. AIE also increased immunoreactivity for HMGB1, which is an agonist of the RAGE receptor, and is released from neurons^60^. HMGB1 plays a key role in ethanol-induced hippocampal proinflammatory gene induction, reduced brain-derived neurotropic factor (BDNF) expression, and loss of neurogenesis^14,34^. Studies of depression-like behavior induced by chronic unpredictable stress indicate that reduced hippocampal neurogenesis and increased depressive-like behaviors are associated with increased proinflammatory cytokines, increased HMGB1, and microglial RAGE expression^54^. We have previously reported that AIE increases HMGB1 in neurons and other brain cells, consistent with overlapping effects of stress and alcohol in many systems^61^. Others have reported RAGE expression on DCX progenitor cells impacts neuronal proliferation^62^. Importantly, although donepezil reversed multiple AIE-induced endpoints, including increased RAGE expression, it did not reverse the AIE-induced increase in HMGB1. AIE persistently increases HMGB1, RAGE, pNFkB p65, and the epigenetic gene silencer, H3K9me2 + IR, associated with decreased BDNF and increased microglial activation. Donepezil reversal of all endpoints except HMGB1, suggests RAGE signaling through pNFkB p65, and the epigenetic gene silencer, H3K9me2 + IR, may be the key mechanisms suppressing neurogenesis.

**Figure 8:**
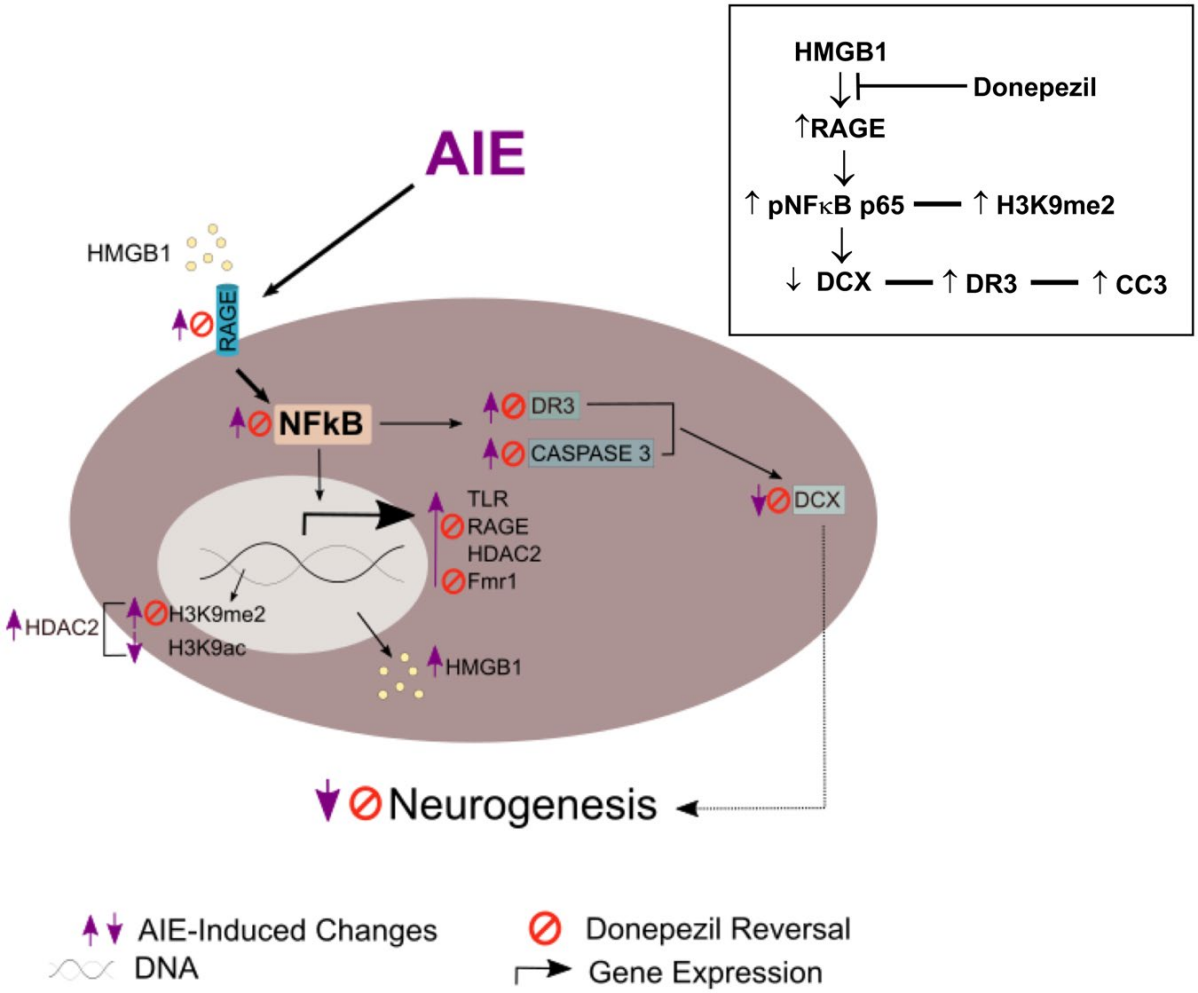
Hypothetical model of AIE-induced adult pathology and the mechanisms of Donepezil reversal of AIE effects. AIE increases expression of adult hippocampal HMGB1 and RAGE consistent with increased signaling of this agonist-receptor combination. HMGB1-RAGE increases *p*NFκB transcription of RAGE feeding forward to enhance expression of DR3, which, in turn activates cell death caspase cascades as indicated by increased active caspase 3, the executioner caspase. RAGE is expressed on DCX + IR neuroprogenitors consistent with RAGE increasing DCX cell death that contributes to the persistent loss of neurogenesis after AIE. AIE increases RAGE, pNFkBp65 and increases H3K9me2, an epigenetic marker of gene silencing. Gene silencing involves histone methylation of H3K9me2 and is reciprocal with deacetylation, the loss of H3K9ac. HDAC2 is increased in neurons by NFkB consistent with reduced BDNF in response to RAGE-NFkB activation. Donepezil reversed RAGE, pNFkBp65, H3K9me2, DR3, caspase-3 and loss of DCX after AIE. These findings are consistent with an anti-inflammatory action of donepezil either through increasing acetylcholine and/or through anti-inflammatory microglial inhibitory mechanisms.

Our observation of AIE-induced increases in DR3-TNFSFR25 and cleaved caspase-3 + IR are consistent with increased HMGB1 promoting activation of NFκB and H3K9me2 through RAGE, which increases proinflammatory gene expression, including the DR3-TNFSRF25 receptor, which is known to activate cell death cascades through the executioner caspase-3. Thus, it is likely that the persistent AIE-induced increase in RAGE activation increases the death of DCX progenitor cells, reducing their maturation to mature dentate granule cell neurons and resulting in reduced neurogenesis (Fig. 8). Together these findings suggest that reversal of the AIE-induced RAGE increase in adulthood by donepezil leads to the down regulation of AIE-induced proinflammatory genes, epigenetic histone modifiers of gene expression, and DR3-caspase cascades, thus restoring neurogenesis. This restoration of DCX + IR weeks after AIE exposure suggests the persistent adult changes following AIE are not necessarily permanent, but rather represent changes in gene expression that can be reversed in adulthood.

We recently reported that an identical treatment regimen with donepezil reversed AIE-induced decreases in dendritic spine density on granule cells in the hippocampal dentate gyrus^12^. In that study, in addition the effects on spine density, AIE also increased expression of the fragile X mental retardation-1 (*Fmr1*) gene and increased *Fmr1* gene H3-K27 acetylation (an epigenetic enhancer of gene expression) and both of those effects were also reversed by donepezil. Those findings gave rise to the intriguing hypothesis that AIE may induce maladaptive alterations in neuronal morphology through specific gene expression and epigenetic mechanisms, which appear sensitive to alteration by systemic donepezil. The present results, also in the dentate gyrus, extend those previous findings and provide additional clues regarding potential mechanisms. FMRP is an RNA binding protein linked to Fragile X-associated mental retardation that represses mRNA translation, including neuronal dendritic mRNA.  It also binds PSD95, a key glutamatergic excitatory synapse protein, consistent with the *Fmr*1 gene regulating hippocampal synapses^63^. In hippocampal neurons, NFkB activation increases excitatory glutamate synapses and decreases inhibitory synapses^64^, as well as increasing proinflammatory gene expression in multiple brain cells^65^. Fragile X-associated mental retardation occurs due to *Fmr1* mutations and increased expression of IL10, a potent anti-inflammatory cytokine^66^, and heterozygotes have been reported to develop immune mediated diseases with Fragile-X disease^67^. Similarly, *Fmr1*-knockout mice have decreased expression of hippocampal proinflammatory cytokine mRNA^68^, consistent with AIE increasing both *Fmr1* and proinflammatory signaling genes. Since AIE increased *Fmr1* expression^12^, it could be that increase is linked to the increases in proinflammatory NFkB gene expression that persist in part due to histone acetylation contributing to lasting increases in expression. The potential linkage between AIE-induced changes in neuroinflammation and epigenetic regulation, as well as *Fmr1* expression and dendritic spine density and morphology, could represent common mechanisms for further study.

Donepezil is an anti-cholinesterase drug that inhibits acetylcholine breakdown and increases its levels in the brain where it is known to be anti-inflammatory^39^. Cholinergic innervation of the hippocampus is known to influence neurogenesis^69^, but has also been shown to inhibit several inflammatory mechanisms, independent of those cholinergic effects. For example, Hwang *et al*.^42^ showed that donepezil directly inhibits proinflammatory gene expression in, and activation of, microglia, in part by inhibiting NFκB signaling^42^. In addition, donepezil is known to attenuate the release of inflammatory factors from microglia and to reduce toxicity mediated by microglia in primary hippocampal neurons^70^. In that same study, *in-vivo* micro-infusion of a pro-inflammatory amyloid-B oligomer into the hippocampal formation resulted in microgliosis and astrogliosis, both of which were attenuated by donepezil. We have previously shown AIE-induced increases in activated astrocytes, and astrocyte-released thrombospondins in the hippocampal formation^35^. Further, previous studies have found that lipopolysaccharide neuroimmune activation can mimic AIE-induced loss of neurogenesis^50^ and that exercise or indomethacin, an anti-inflammatory drug, can block both the AIE-induced increase in neuroimmune gene expression and the loss of adult neurogenesis^50,57^. Thus, the reversal of AIE-induced pNFκB increases by donepezil is consistent with an anti-inflammatory action of donepezil through its anti-cholinesterase and/or other anti-inflammatory mechanisms. Whether donepezil reverses the presently observed AIE-induced changes through its cholinergic or non-cholinergic^70^ actions remains unclear. Future studies will be designed to address that question. In addition, the sub-chronic donepezil dosing schedule that we used in the present study does not allow us to determine whether the observed reversal effects were due to acute vs. sub-chronic donepezil effects. Clearly, this limits the interpretation of the findings, but since donepezil is used chronically or sub-chronically in clinical applications, our findings are translationally relevant.

Emerging studies link epigenetic and neuroimmune signaling mechanisms to long lasting changes in brain gene expression, plasticity, and pathology. In general, methylation and acetylation of histones and DNA have been found to regulate suppresion and induction of gene expression. Neuronal differentiation involves sequential reductions in gene expression through increased methylation and reduced histone acetylation as dividing stem cells differentiate into cells primarily expressing mature neuronal genes. Neuroimmune signaling across neurons and glia also contributes to brain development through processes such as axon guidance and activity dependent synapse development^71^. We found H3K9me2 + IR, a histone epigenetic gene silencing marker, was increased in the adult dentate gyrus after AIE. Although this single epigenetic finding is not sufficient to argue for a specific epigenetic mechanism underlying AIE-induced hippocampal alterations, it is consistent with earlier studies indicating that AIE decreased hippocampal H3K9 acetylation in association with decreased neurogenesis^14^, since increased methylation and decreased acetylation are linked and both reduce gene expression. AIE-induced histone modifications have been previously studied in amygdala, where AIE was found to increase the expression of histone deacetylase 2 (HDAC2), a deacetylase that reduced H3K9 acetylation, expression of BDNF (particularly Exon IV), activity-regulated cytoskeleton-associated (Arc) protein, and dendritic spine density^58^. Increased HDAC2 decreases histone acetylation of BDNF exon IV reducing BDNF protein, and is associated with persistent adult anxiety and increased consumption and preference for ethanol^58^. These persistent AIE-induced increases in anxiety and ethanol preference were reversed by treatment with the histone deacetylase (HDAC) inhibitor, trichostatin A (TSA). Similarly, we have previously reported that AIE-induced decreases in doublecortin IR in the dentate gyrus were reversed by TSA in association with a reversal of AIE-induced histone changes and reduced hippocampal BDNF expression^14^. Thus, the persistent AIE-induced decrease in adult neurogenesis is linked to epigenetic histone repression of BDNF, as well as to persistent increases in neuroimmune gene signaling. To our knowledge, donepezil is not known to directly alter HDAC activity, although our findings suggest it reverses both increased pNFκBp65 and increased H3K9me2 after AIE, consistent with these changes being linked to the persistent loss of adult neurogenesis in the hippocampal formation.

Recent studies have reported that NFκB activation in cortical neurons induces HDAC2, which is associated with suppression of synapses, synaptic plasticity and cognition^72^, which have also been reported after AIE. These findings are consistent with HMGB1 activation of RAGE, or a TLR increasing expression of proinflammatory genes through NFκB, as well as with increased HDAC2 expression blunting trophic and synapse gene expression and contributing to a long-lasting reduction in neurogenesis (Fig. 8). As noted above, we have also found that exercise or anti-inflammatory drugs block AIE-induced neuroimmune activation of proinflammatory genes, pNFκB expression, and inhibition of neurogenesis; and that HDAC inhibitors increase BDNF and inhibit neuroimmune gene induction^14^. Exercise has also been reported to increase BDNF expression through reduced histone acetylation of BDNF exon IV, decreased HDAC expression, and increases in BDNF mRNA^73^. These findings are all consistent with AIE shifting hippocampal gene expression toward proinflammatory signaling and blunted trophic activity, which are reversed by exercise, anti-inflammatory drugs and anti-inflammatory/pro-trophic HDAC inhibitors (see^74^). Thus, it is likely that the mechanisms underlying our observation that donepezil reversed AIE-induced inhibition of neurogenesis include the reversal of RAGE induction, increased pNfκBp65 and H3K9me2 regulators of gene expression, consistent with AIE induction of persistent RAGE. Future studies will further explore these possible mechanisms.
